# Hybrid side-by-side and stent-in-stent deployment of three slim multi-hole metallic stents for malignant hilar biliary obstruction

**DOI:** 10.1055/a-2724-8115

**Published:** 2025-11-06

**Authors:** Soma Fukuda, Masato Endo, Yusuke Niisato, Yuya Hagiwara, Hirotaka Uno, Taku Sakamoto, Kiichiro Tsuchiya

**Affiliations:** 1 38515 Department of Gastroenterology, Institute of Medicine, University of Tsukuba, Ibaraki, Japan; 2 90521 Graduate School of Comprehensive Human Sciences, University of Tsukuba, Ibaraki, Japan

Malignant hilar biliary obstruction (MHBO), especially Bismuth types III and IV, often requires trisegment drainage. Triple uncovered self-expandable metal stent (UCSEMS) placement with the stent-in-stent (SIS) method has been reported but is technically demanding 1 . A hybrid side-by-side (SBS) plus SIS approach has also been described, yet tumor ingrowth remains problematic with UCSEMSs 2 3 . Recently, slim 6-mm multi-hole SEMSs (MHSEMSs) (HANAROSTENT Biliary Multi-Hole Benefit; M.I. Tech Co., Ltd., Pyeongtaek, South Korea) featuring 1.5-mm side holes along the circumference have been introduced ( Fig. 1 ). These stents have been applied for SBS and SIS 4 5 . However, trisegment drainage with MHSEMSs remains technically challenging. We present the first case of trisegment drainage using a hybrid method with three slim MHSEMSs.

**Fig. 1:**
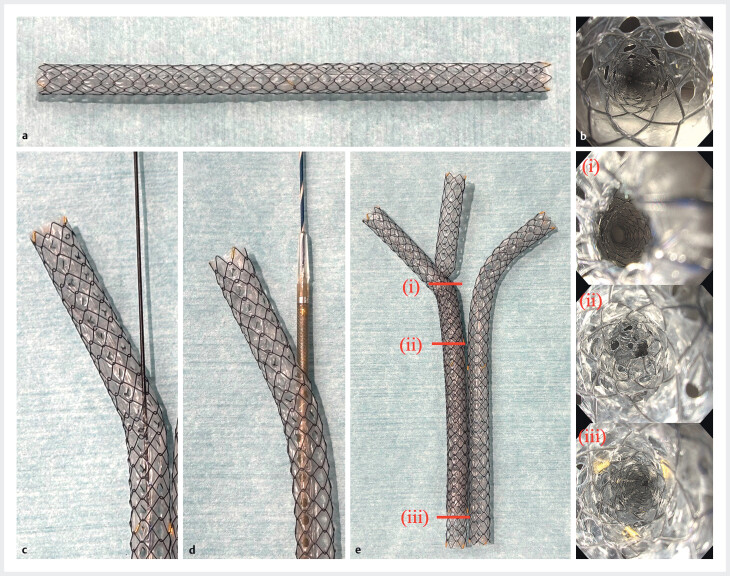
**a** The 6-mm slim multi-hole SEMS (MHSEMS), which is covered and equipped with multiple 1.5-mm side holes arranged along the circumference. **b** Endoscopic view of the stent lumen. **c** A guidewire passing through a side hole of the stent. **d** A 5.9-Fr delivery sheath advanced through a side hole. **e** Hybrid method (side-by-side and stent-in-stent) using three slim MHSEMSs. (i–iii) Endoscopic views of the stent lumen after stent-in-stent deployment.

A 65-year-old woman with cecal cancer and liver metastasis developed cholangitis after plastic stents had been placed in the left hepatic duct and right anterior branch. Imaging showed disease progression with occlusion of these stents and new dilatation of the isolated right posterior branch ( Fig. 2 ). Because trisegment drainage was required, endoscopic drainage with three 6-mm MHSEMSs was performed.

**Fig. 2:**
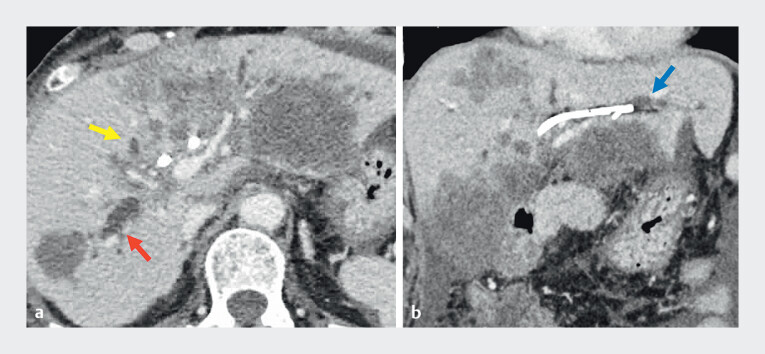
Contrast-enhanced CT showing progression of liver metastases with intrahepatic bile duct dilatation (yellow arrow, right anterior branch; red arrow, right posterior branch; blue arrow, left hepatic duct). **a** Axial view. **b** Coronal view.

After stent removal, guidewires were placed in the left, right anterior, and right posterior ducts ( Fig. 3 ). Two MHSEMSs were deployed simultaneously in the left and right posterior ducts using the SBS technique. A guidewire was successfully advanced into the right anterior branch through a small side hole, enabling SIS placement of the third MHSEMS ( Fig. 4 , Video 1 ). Final cholangiography confirmed successful trisegment drainage without adverse events ( Fig. 5 ).

**Fig. 3:**
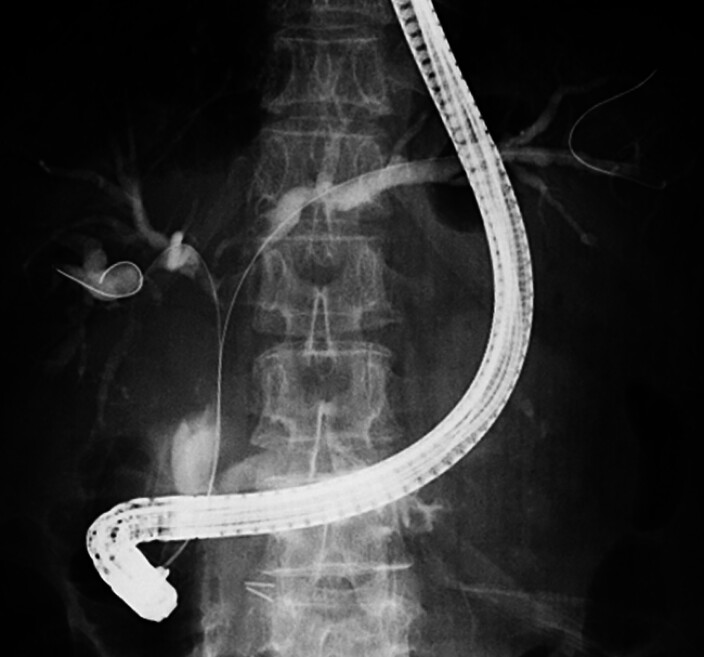
ERCP demonstrating a Bismuth type IV hilar stricture with isolated left hepatic duct, right anterior, and right posterior branches.

**Fig. 4:**
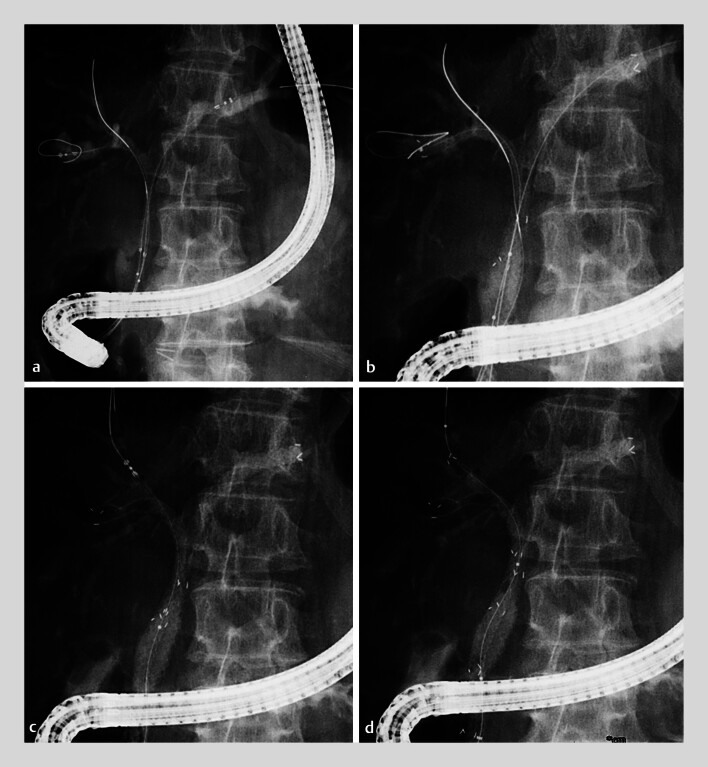
Hybrid deployment procedure. **a** Two delivery systems positioned in the left hepatic duct and right posterior branch. **b** Simultaneous deployment of two MHSEMSs using the side-by-side (SBS) technique. **c** Positioning of the third MHSEMS delivery system through a side hole of the posterior stent. **d** Deployment of the third MHSEMS using the stent-in-stent (SIS) technique.

Hybrid side-by-side plus stent-in-stent deployment of three slim multi-hole SEMSs demonstrates an effective option for malignant hilar obstruction requiring trisegment drainage.Video 1

**Fig. 5:**
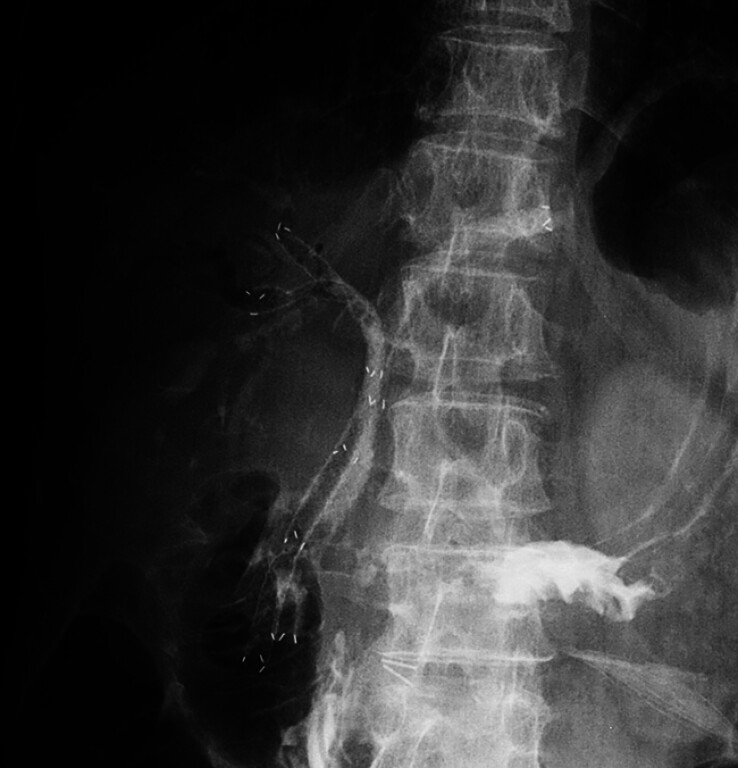
Completion of hybrid SBS and SIS deployment of three slim MHSEMSs with good contrast drainage.

This case highlights successful trisegment drainage using a hybrid method with three slim MHSEMSs. The multi-hole design allowed guidewire passage and branch drainage, while the slim delivery system enabled simultaneous SBS deployment. Compared with plastic stents, longer patency is expected, and UCSEMS-related ingrowth risk may be reduced. This hybrid approach offers a technically easier and effective option for malignant hilar obstruction requiring trisegment drainage.

Endoscopy_UCTN_Code_TTT_1AR_2AZ

## Notes

Endoscopy E-Videos https://eref.thieme.de/e-videos
*E-Videos* is an open access online section of the journal *Endoscopy* , reporting on interesting cases and new techniques in gastroenterological endoscopy. All papers include a high-quality video and are published with a Creative Commons CC-BY license. Endoscopy E-Videos qualify for HINARI discounts and waivers and eligibility is automatically checked during the submission process. We grant 100% waivers to articles whose corresponding authors are based in Group A countries and 50% waivers to those who are based in Group B countries as classified by Research4Life (see: https://www.research4life.org/access/eligibility/ ). This section has its own submission website at https://mc.manuscriptcentral.com/e-videos .
